# An artificial intelligence-assisted diagnostic system for the prediction of benignity and malignancy of pulmonary nodules and its practical value for patients with different clinical characteristics

**DOI:** 10.3389/fmed.2023.1286433

**Published:** 2023-12-22

**Authors:** Lichuan Zhang, Yue Shao, Guangmei Chen, Simiao Tian, Qing Zhang, Jianlin Wu, Chunxue Bai, Dawei Yang

**Affiliations:** ^1^Department of Respiratory Medicine, Affiliated Zhongshan Hospital of Dalian University, Dalian, China; ^2^Department of Pulmonary and Critical Care Medicine, Zhongshan Hospital Fudan University, Shanghai, China; ^3^Department of Pulmonary and Critical Care Medicine, Zhongshan Hospital (Xiamen), Fudan University, Xiamen, China; ^4^Shanghai Respiratory Research Institution, Shanghai, China

**Keywords:** artificial intelligence (AI), pulmonary nodules, benign and malignant, Chest CT, clinical characteristics

## Abstract

**Objectives:**

This study aimed to explore the value of an artificial intelligence (AI)-assisted diagnostic system in the prediction of pulmonary nodules.

**Methods:**

The AI system was able to make predictions of benign or malignant nodules. 260 cases of solitary pulmonary nodules (SPNs) were divided into 173 malignant cases and 87 benign cases based on the surgical pathological diagnosis. A stratified data analysis was applied to compare the diagnostic effectiveness of the AI system to distinguish between the subgroups with different clinical characteristics.

**Results:**

The accuracy of AI system in judging benignity and malignancy of the nodules was 75.77% (*p* < 0.05). We created an ROC curve by calculating the true positive rate (TPR) and the false positive rate (FPR) at different threshold values, and the AUC was 0.755. Results of the stratified analysis were as follows. (1) By nodule position: the AUC was 0.677, 0.758, 0.744, 0.982, and 0.725, respectively, for the nodules in the left upper lobe, left lower lobe, right upper lobe, right middle lobe, and right lower lobe. (2) By nodule size: the AUC was 0.778, 0.771, and 0.686, respectively, for the nodules measuring 5–10, 10–20, and 20–30 mm in diameter. (3) The predictive accuracy was higher for the subsolid pulmonary nodules than for the solid ones (80.54 vs. 66.67%).

**Conclusion:**

The AI system can be applied to assist in the prediction of benign and malignant pulmonary nodules. It can provide a valuable reference, especially for the diagnosis of subsolid nodules and small nodules measuring 5–10 mm in diameter.

## Introduction

Bronchogenic carcinoma, simply referred to as lung cancer, remains the leading cause of cancer deaths for both males and females according to Global Cancer Statistics 2023 (1). Adenocarcinoma is more common in the Asian population, particularly never-smokers (2, 3). Approximately 75% of lung cancer cases are diagnosed at advanced or late stages (4). Missing the optimal timing of surgical removal usually results in poor prognosis. On the other hand, the 5-year survival rate for early-stage non-small cell lung cancer is above 50% (5), and the 10-year survival rate for Stage I lung cancer with surgical treatment reaches as high as 92% (6). Therefore, improving the early diagnosis rate is crucial for prolonging lung cancer survival.

As CT imaging technology has rapidly developed, low-dose computed tomography (LDCT) has become an increasingly accepted method for lung cancer screening (7). However, challenges remain, as radiologists are faced with a high demand for clinical testing. Furthermore, a standardized diagnosis of pulmonary nodules (early-stage lung cancer) is various among different countries, areas, and hospitals, due to heterogeneous of biological and healthcare economics policy, especially during and post the global pandemic of COVID-19 (8). To address this issue, it is necessary to provide training based on large-scale imaging data. The concept of Artificial Intelligence (AI), coined at the Dartmouth Conference in 1956 (9), refers to the simulation of intelligent behavior by computers with minimal human intervention (10). Recent years have witnessed theoretical and practical advances in AI, such as deep learning (DL), and their applications in different fields of medical data analysis (11). Among these, the AI-assisted diagnostic system for pulmonary nodules (referred to as “the AI system” hereinafter) is becoming increasingly mature. By applying an effective extraction of the imaging characteristics of malignant nodules, the AI system can realize the automatic and accurate detection of small pulmonary nodules, as well as the assessment of malignancy risk (12). Not only does AI improve the efficiency of medical image reading, but it also enhances the accuracy rate of diagnosis, reaching over 90% (13). With regard to the application of AI as assistive technology for the judgment of benignity or malignancy of pulmonary nodules in the real world, little research is available concerning which subgroup(s) with which clinical characteristics may affect the predictive accuracy of the AI model. This study aimed to apply an AI-assisted system in the predictive analysis of pulmonary nodules, including automatic detection of nodules, segmentation of lesions, measurement of imaging parameters, and differentiation between benign and malignant nodules. We evaluated the diagnostic effectiveness of the AI model, and discussed its practical value for subgroups with different clinical characteristics, in order to make optimal use of AI in clinical diagnoses.

## Methods

### Study design and data source

**Figure 1:**
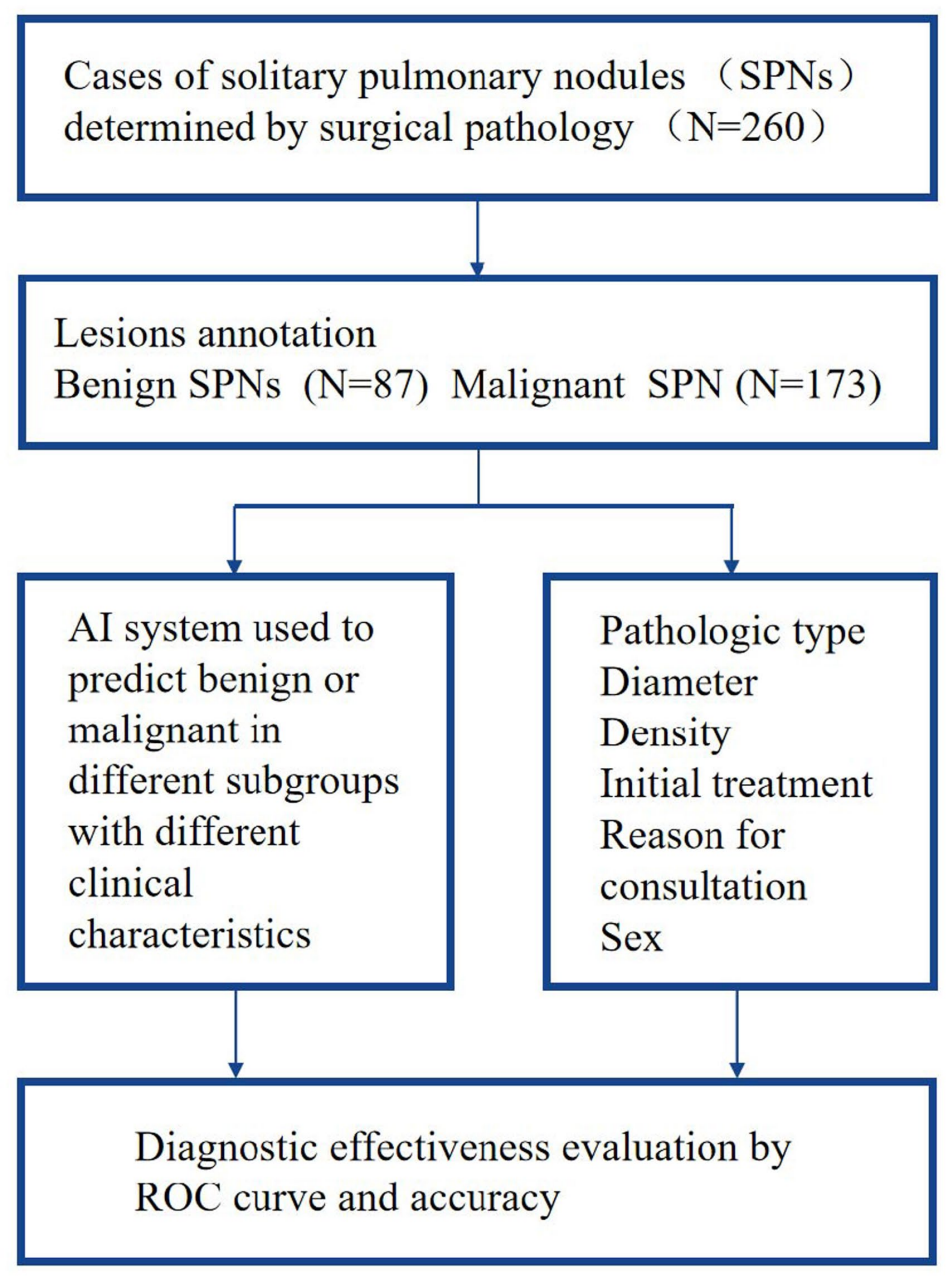
The flow chart analysis workflow of the collected dataset.

This retrospective study used data for pulmonary nodules managed using surgical treatment during the period between January 2018 and April 2021 at the Affiliated Zhongshan Hospital of Dalian University. The study was approved by their Ethical Board and exempted from informed consent. The criteria for data inclusion were: (1) a definitive diagnosis based on surgical pathology, (2) a normal CT scan of the chest taken before the surgery, and a clear, qualified thin-slice image (thickness of 1.00 mm) being available, (3) at least one pulmonary nodule being present per case, (4) nodules measuring 5–30 mm in diameter, and (5) complete and detailed clinical information on the patient. Among the 260 cases included in the dataset, a total of more than 260 SPNs were identified by the clinical CT evaluation, but in each case only one nodule was surgically removed and consequently confirmed as being malignant or benign according to surgical pathology. Malignant nodules accounted for 66.54% of the dataset (173 cases) and benign nodules, 33.46% (87 cases). Males accounted for 41.54% of the dataset (108 cases) and females, 58.46% (152 cases). The age range was from 26 to 83 years. The analysis workflow of the collected dataset is showed as a flow chart in Figure 1.

### Instruments and examinations

#### CT examination

A multi-slice spiral CT scan of the chest was applied to each patient, using a Siemens SOMATOM Definition CT scanner (64-slice or above). The patient was required to lie in a supine position, take a deep breath in and hold it during the CT scan, which ranged from the apex to the base of the lung. The technical parameters of the routine CT scan were: tube voltage 100–120 kV, tube current 100–350 mAs, scan slice thickness 5.0–8.0 mm, slice spacing 4.0–6.0 mm, and matrix size 512 × 512. Subsequently, thin-slice reconstruction (thickness of 1.0 mm) was performed using the built-in software.

#### AI identification

**Figure 2:**
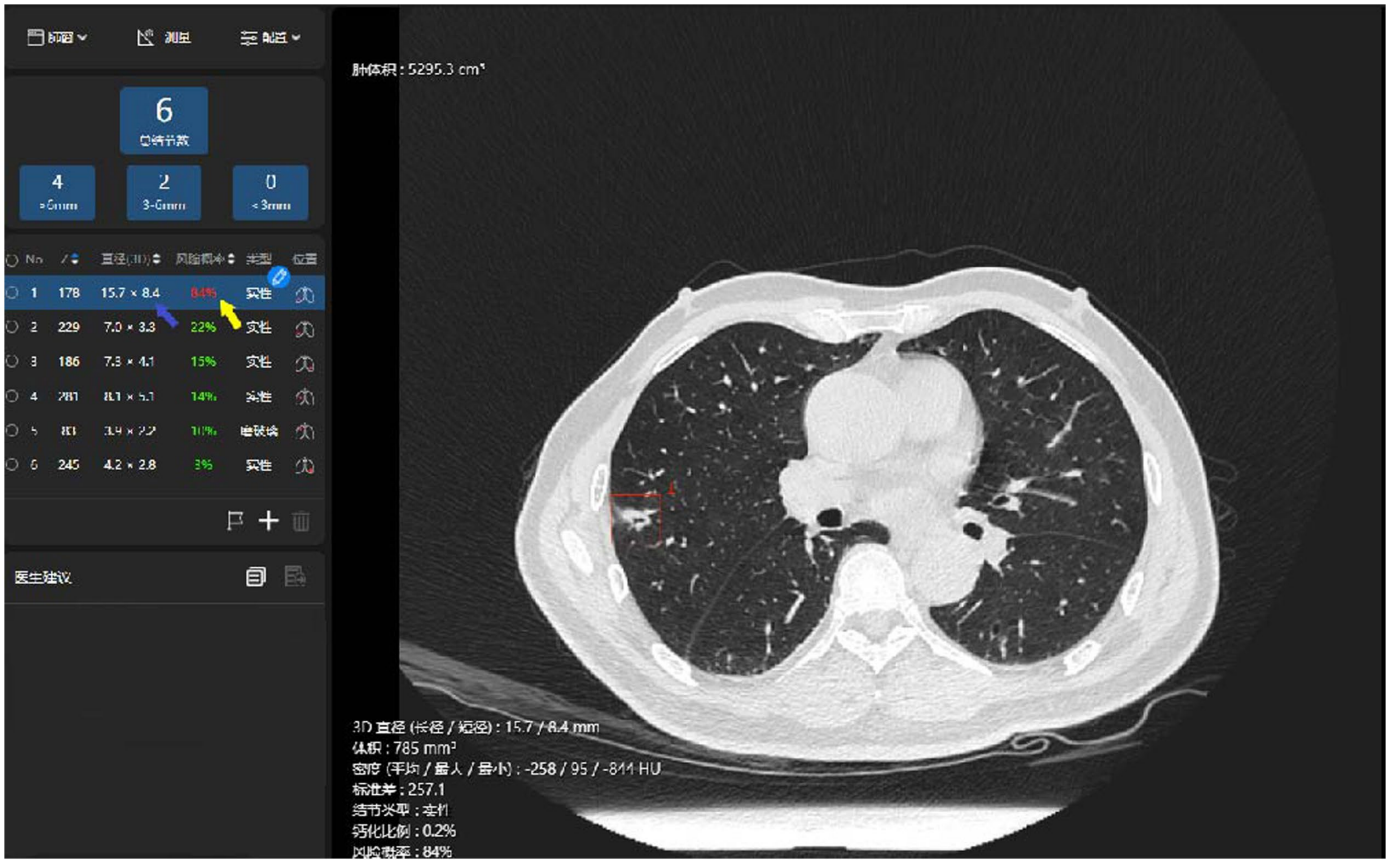
Illustration of the AI identification of pulmonary malignant nodules. The system has identified a nodule in the thin-slice chest CT imaging (the red box), automatically measured its long- and short-axis diameters in 3D (the blue arrow), and prompted a prediction of malignancy risk (the yellow arrow).

**Figure 3:**
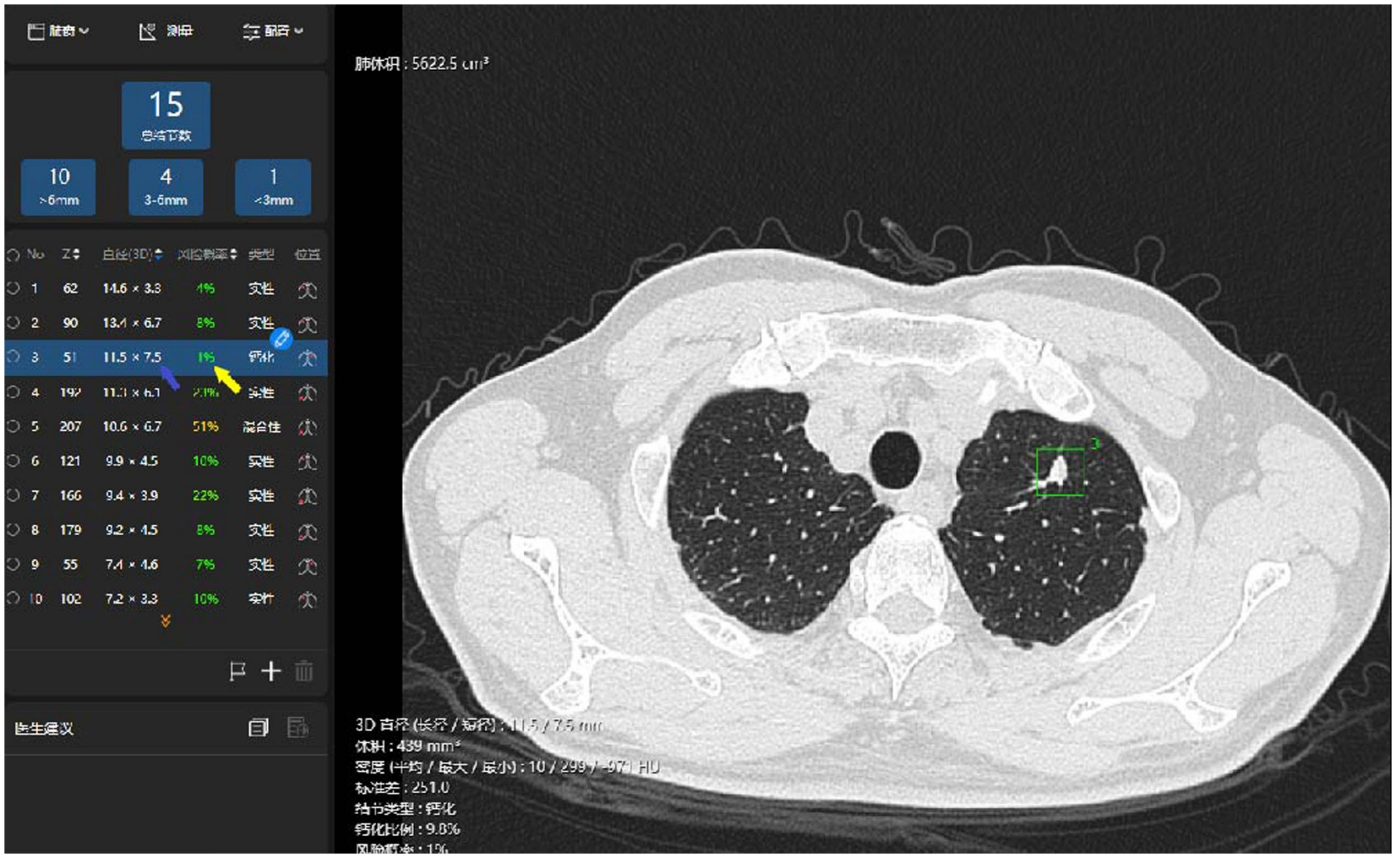
Illustration of the AI identification of pulmonary benign nodules. The system has identified a nodule in left upper lobe superior segment (the red box), automatically measured its long- and short-axis diameters in 3D (the blue arrow), and prompted a prediction of malignancy risk (the yellow arrow).

The thin-slice chest CT imaging data were imported into the AI system (σ-Discover/Lung, V1.0.2, 12 Sigma Technologies, United States) for automatic detection of the pulmonary nodules and predictive analysis of benignity or malignancy (14). The system recorded the number of nodules, position, long-axis diameter, and short-axis diameter, and produced a prediction of malignancy risk (Figures 2, 3), which was completely calculated based on the CT image of the nodules, without reference to the patient’s clinical information. The system leverages deep learning, also referred to as deep neural network (DNN), which is a neural network architecture integrating multiple hidden layers. The deep convolutional neural network (DCNN) enables it to implement 3D detection, 3D segmentation, and 3D analysis of the pulmonary nodules (15, 16). According to the previous training and validation of the model using a local dataset (17), if an AI outcome is ≥65%, the nodule is predicted to be malignant; the higher the value, the more likely it is to be malignant. Conversely, an AI outcome <65% means the prediction is for benignity.

### Statistical analyses

IBM SPSS 20.0 software was applied for statistical analysis in this study. We considered a positive result according to surgical pathology to be the “gold standard” for the diagnosis of pulmonary nodules, following the pathologic diagnostic criteria for lung cancer specified by the World Health Organization (WHO) (17, 18). We examined the results of the DL algorithm-based prediction model for nodule benignity and malignancy, and analyzed the differences in diagnostic effectiveness between the clinical subgroups by calculating the accuracy, sensitivity, specificity, positive predictive value, and negative predictive value. The AI contributed to the judgment of benign and malignant, showing certain value in early diagnosis of lung cancer (12, 19).

To be more specific, the accuracy was expressed as the ratio of the number of correctly predicted nodules to the total number of nodules; the sensitivity, or true positive rate (TPR), was expressed as the ratio of the number of malignant nodules correctly predicted to the total number of malignant nodules; the specificity, or true negative rate (TNR), was expressed as the ratio of the number of benign nodules correctly predicted to the total number of benign nodules; the positive predictive value (PPV) was expressed as the ratio of the number of malignant nodules correctly predicted to the number of malignant nodules correctly predicted and benign nodules incorrectly predicted as malignant; and the negative predictive value (NPV) was expressed as the ratio of the number of benign nodules correctly predicted to the number of benign nodules correctly predicted and malignant nodules incorrectly predicted as benign. Continuous data with normal distributions are presented as the mean and SD, whereas those not normally distributed are presented as the median and IQR after assessing normality by the Shapiro–Wilk test. An ROC curve was created to evaluate the performance of the AI model in the prediction of benign and malignant pulmonary nodules, and its diagnostic effectiveness was expressed by the AUC. A chi-squared test (α = 0.05) was used for comparison between the groups, and *p* < 0.05 was considered to be statistically significant.

## Results

### Demographics and imaging characteristics

The 260 cases of pulmonary nodules with surgical treatment were classified into benign and malignant groups according to the pathologic results, and demographic features, clinical manifestations, and imaging characteristics. The pathologic diagnostic results for the two groups are shown in Table 1. There were no significant differences between the benign and malignant groups concerning mean age, sex, reason for consultation, but the differences were statistically significant concerning mean nodule diameter (*p* = 0.002), nodule density (*p* = 0.011), and nodule position (*p* = 0.045).

**Table 1:** Demographics and imaging characteristics.

Characteristics	Number	Benign group	Malignant group	*p* value
Patient	260	87 (33.46%)	173 (66.54%)	
Age (mean ± standard deviation)	58.88 ± 10.18	58.72 ± 1.06	58.96 ± 0.79	0.882
Sex				
Female	152 (58.46%)	48 (55.17%)	104 (60.12%)	
Male	108 (41.54%)	39 (44.83%)	69 (39.88%)	0.591
Reason for consultation (with or without symptoms)				
Physical examination	204 (78.46%)	72 (82.76%)	132 (76.3%)	
Appearance of symptoms	56 (21.54%)	15 (17.24%)	41 (23.7%)	0.879
Nodule		27 (13.5%)	173 (86.5)	
Diameter, mm (mean ± standard deviation)	14.69 ± 0.40	12.99 ± 0.64	15.54 ± 0.50	0.002
Nodule density				
Solid nodule	111 (42.69%)	44 (51.76%)	67 (38.29%)	
Subsolid nodule	149 (57.31%)	41 (48.24%)	108 (61.71%)	0.011
Pure ground-glass nodules	123 (82.55%)	31 (75.61%)	92 (85.19%)	
Mixed-ground glass nodules	26 (17.45%)	10 (24.39%)	16 (14.81%)	0.608
Nodule position				
Right upper lobe	84 (32.31%)	16 (18.39%)	68 (39.31%)	
Right middle lobe	20 (7.69%)	12 (13.79%)	8 (4.62%)	
Right lower lobe	53 (20.38%)	29 (33.33%)	24 (13.87%)	
Left upper lobe	58 (22.31%)	18 (20.69%)	40 (23.12%)	
Left lower lobe	45 (17.31%)	12 (13.80%)	33 (19.08%)	0.045
Pathologic type				
Benign nodule	87			
Carbon dust deposition	4 (4.60%)			
Hamartoma	11 (12.64%)			
Fibrous tissue hyperplasia	11 (12.64%)			
Granulomatous inflammation	15(17.24%)			
Inflammatory lesion	38 (43.68%)			
Tuberculosis	5 (5.75%)			
Intrapulmonary lymph node	3 (3.45%)			
Malignant nodule	173			
AAH	6 (3.47%)			
AIS	33 (19.08%)			
MIA	20 (11.56%)			
IA	107 (61.85%)			
Squamous cell carcinoma	4 (2.31%)			
Mucinous adenocarcinoma	3 (1.73%)			

### AI prediction results for the 260 pulmonary nodules

**Figure 4:**
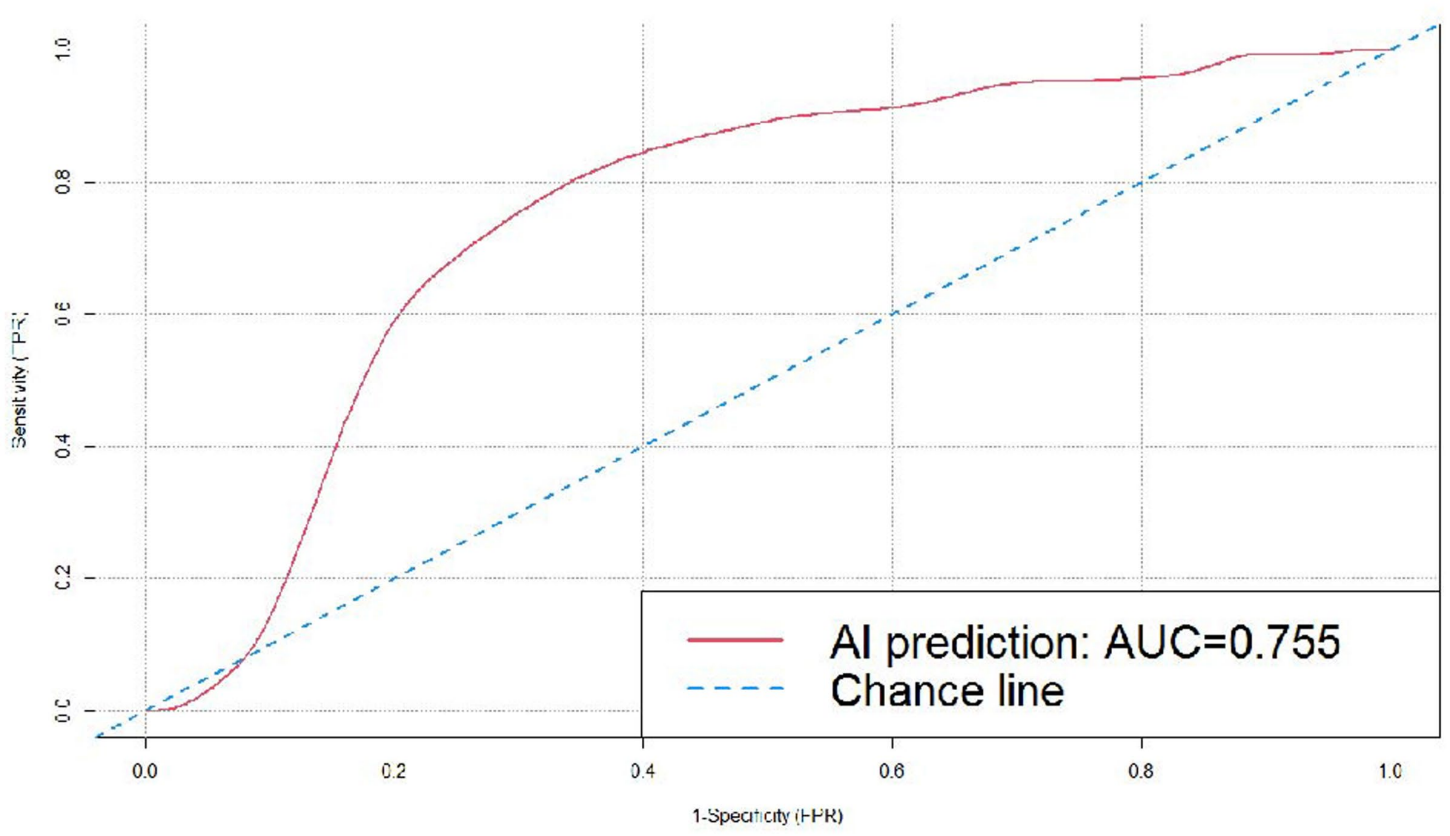
ROC curve for the DL algorithm-based model in the prediction of benignity and malignancy of the pulmonary nodules.

The dataset included 173 malignant cases, among which adenocarcinoma was the major pathologic type, accounting for 169 cases, with the remaining four cases being squamous cell carcinoma. The cases of adenocarcinoma included atypical adenomatous hyperplasia (AAH), adenocarcinoma *in situ* (AIS), minimally invasive adenocarcinoma (MIA), invasive adenocarcinoma (IA), and mucinous adenocarcinoma. The 87 benign cases included inflammatory pseudotumor, carbon dust deposition, hamartoma, fibrous tissue hyperplasia, granulomatous inflammation, inflammatory disease, tuberculoma, and intrapulmonary lymph nodes (Table 1). In this study, the AI software detected 100% of the pulmonary nodules, and we further examined the prediction results for benign and malignant nodules (Table 2). Among the 173 malignant nodules, 155 cases (89.60%) were correctly predicted, and 18 cases (10.40%) were incorrectly predicted as being benign. The AI predictive accuracy was 77.57%, with a sensitivity of 89.60%, and a specificity of 48.28%. PPV and NPV were 77.50 and 70.00%, respectively, and the AUC was 0.755 (Figure 4).

**Table 2:** AI prediction results for the 260 pulmonary nodules.

Pathology	Correct prediction (number)	Incorrect prediction (number)	Total
Malignant nodule	155	18	173
Benign nodule	42	45	87
Total	197	63	260

^*^p < 0.05.

### AI prediction results according to anatomical position, nodule density, and nodule diameter

**Figure 5:**
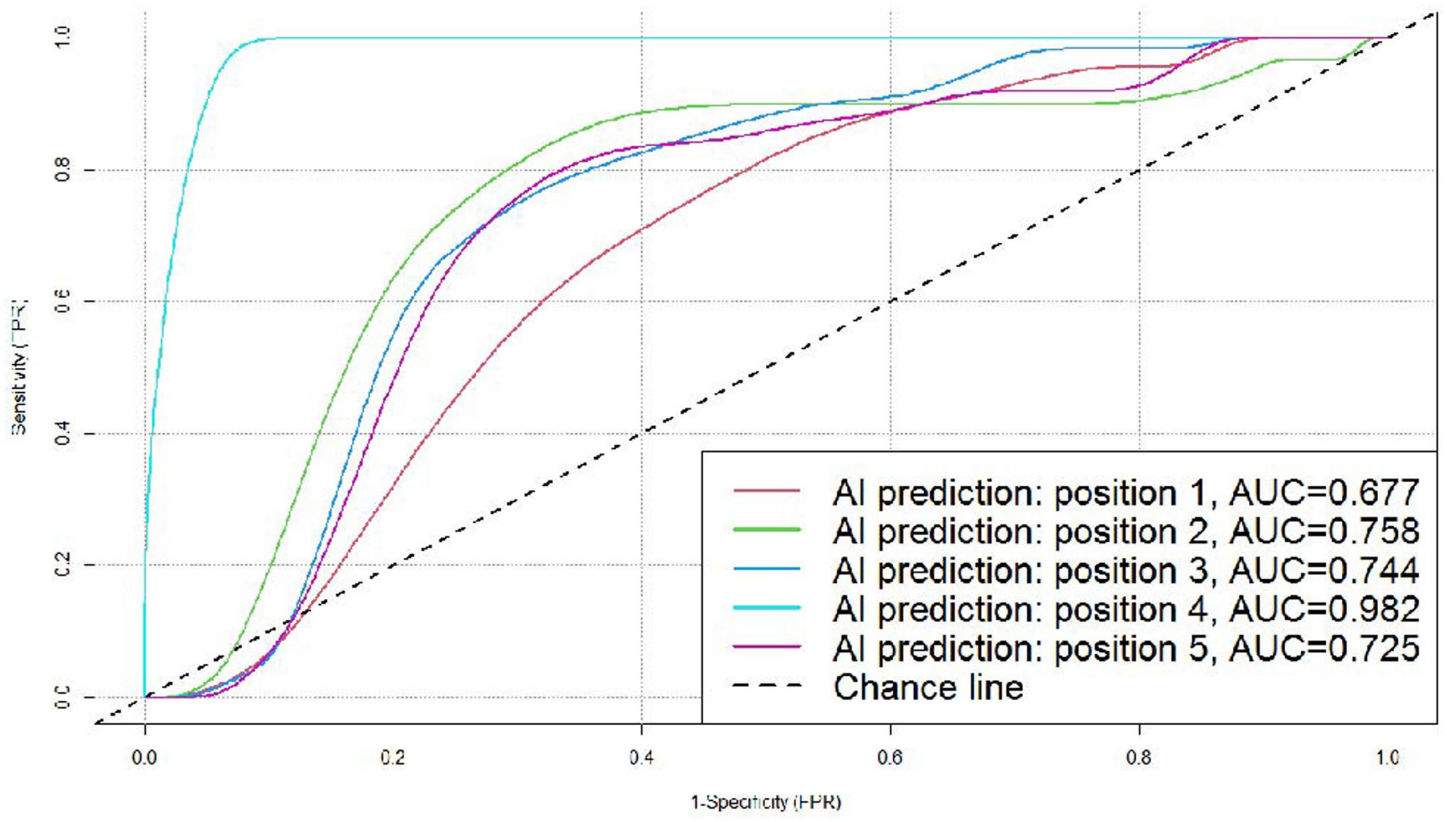
ROC curves for the DL algorithm-based model in the prediction of benignity and malignancy of pulmonary nodules in different positions. Position 1: left upper lobe; position 2: left lower lobe (all malignant, not included in this figure); position 3: right upper lobe; position 4: right middle lobe; and position 5: right lower lobe.

In the subgroups according to the position of the nodules, the AI system correctly predicted 43 (37 malignant and 6 benign) out of the 58 nodules in the left upper lobe, 38 (30 malignant and 8 benign) out of the 45 nodules in the left lower lobe, 68 (60 malignant and 8 benign) out of the 84 nodules in the right upper lobe, 16 (eight malignant and eight benign) out of the 20 nodules in the right middle lobe, and 32 (20 malignant and 12 benign) out of the 53 nodules in the right lower lobe. We created ROC curves by calculating TPR and FPR using different threshold values, and the AUC was 0.677, 0.758, 0.744, 0.982, and 0.725, respectively, for the nodules in the left upper lobe, left lower lobe, right upper lobe, right middle lobe, and right lower lobe, which demonstrated that the AI system had fairly good diagnostic effectiveness for these subgroups, especially for nodules in the right middle lobe (Figure 5).

In the subgroups according to nodule density, the AI system correctly predicted 74 (55 malignant and 19 benign) out of the 111 solid nodules, and 120 (99 malignant and 21 benign) out of the 149 subsolid nodules. The subsolid nodules included 123 pure ground-glass nodules (pGGNs) and 26 mixed-ground glass nodules (mGGNs), the AI system correctly predicted 100 (84 malignant and 16 benign) out of the 123 pGGNs, and 20 (15 malignant and 5 benign) out of the 26 mGGNs. As shown in Table 3, the AI software performed better in the prediction of the subsolid nodules than the solid ones, showing a statistically significant difference (*p* < 0.05), but there no significant differences between the pGGNs and mGGNs groups.

**Table 3:** Comparison of the AI prediction results divided by nodule density.

Subgroup	Sensitivity	Specificity	PPV	NPV	Accuracy
Divided by nodule density					
Solid nodule (*n* = 111)	82.08%	43.18%	68.75%	61.29%	66.67%*
Subsolid nodule (*n* = 149)	93.40%	51.22%	83.19%	70.00%	80.54%*

**Figure 6:**
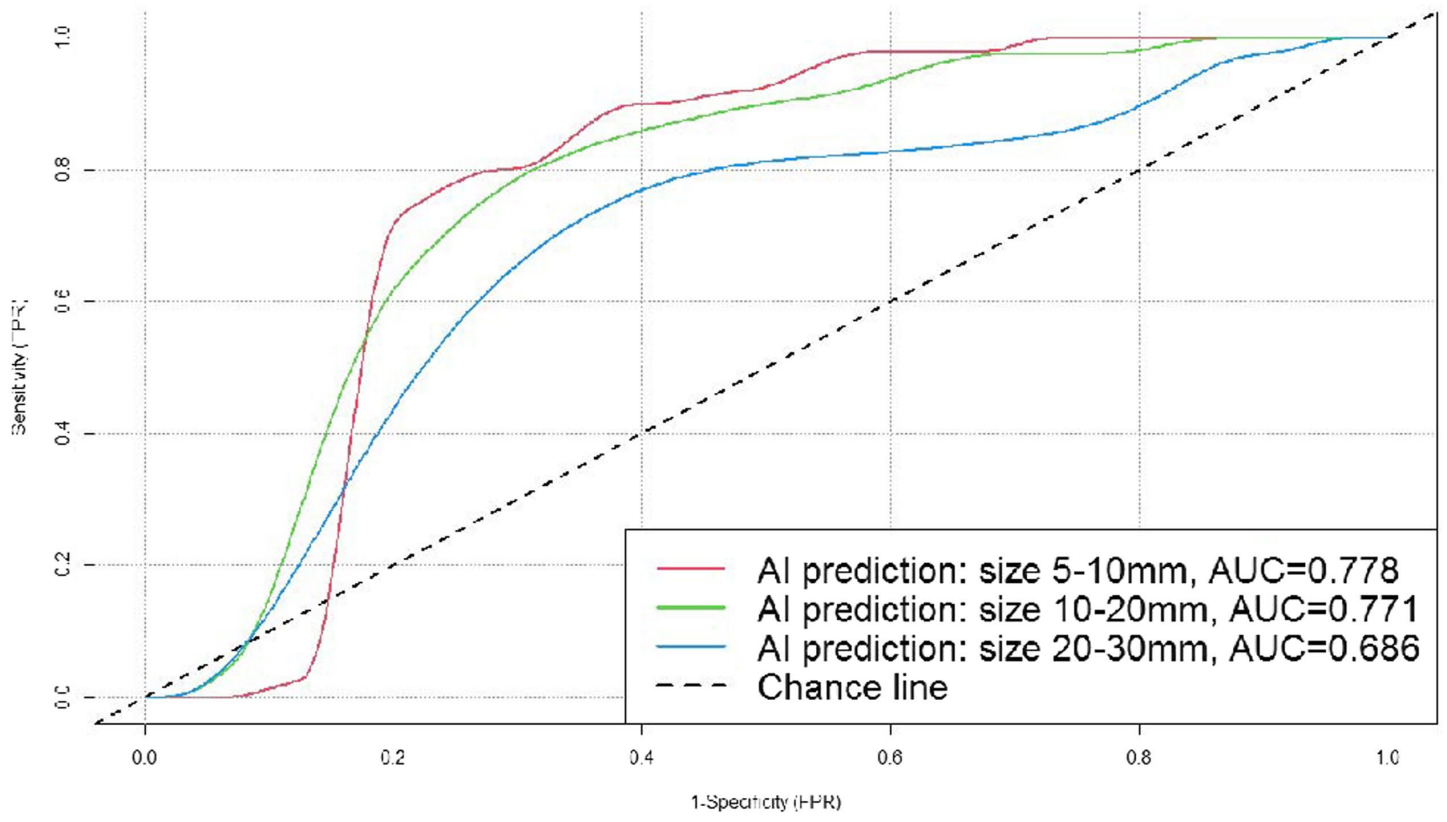
ROC curves for the DL algorithm-based model in the prediction of benignity and malignancy of pulmonary nodules sizes.

All the pulmonary nodules included in the dataset measured 5–30 mm (both inclusive) in diameter. Stratification by nodule diameter showed that the AI system correctly predicted 91 (76 malignant and 15 benign) out of the 118 nodules measuring 5–10 mm (both inclusive) in diameter, 67 (27 malignant and 20 benign) out of the 90 nodules measuring 10–20 mm (20 mm inclusive) in diameter, and 39 (32 malignant and 7 benign) out of the 42 nodules measuring 20–30 mm (30 mm inclusive) in diameter. We created ROC curves by calculating TPR and FPR using different threshold values, and the AUC was 0.778, 0.771, and 0.686 (Figure 6), respectively, for the three subgroups, which demonstrated that the AI system had fairly good diagnostic effectiveness for the pulmonary nodules measuring 5–30 mm in diameter, and especially for those of 5–10 mm (both inclusive) in diameter.

### AI prediction results divided by initial treatment applied, reason for consultation, and sex

We noted three types of treatment processes that the patients experienced after the identification of pulmonary nodules: in some cases, an empirical anti-infection treatment (levofloxacin 500 mg/day or moxifloxacin 400 mg/day for 7–10 days) was administered before the surgery (6–14 days of anti-inflammatory treatment); in other cases, no empirical anti-infection treatment was administered and only follow-ups (for 1–6 months) were arranged before the surgery; and the third subgroup received immediate surgical treatment. The AI system correctly predicted 43 (37 malignant and 6 benign) out of the 60 nodules with anti-inflammatory treatment before surgery, 114 (84 malignant and 30 benign) out of the 146 nodules without anti-inflammatory treatment but with follow-ups before surgery, and 39 (34 malignant and 5 benign) out of the 54 nodules with immediate surgical treatment, showing no significant difference (*p* > 0.05) in the predictive accuracy.

In the subgroups according to reason for consultation, the AI system correctly predicted 155 (122 malignant and 33 benign) out of the 204 nodules identified in chest CT for physical examinations, and 42 (33 malignant and 9 benign) out of the 56 nodules identified in chest CT after the appearance of symptoms, showing no significant difference (*p* > 0.05) in the predictive accuracy (Table 4).

**Table 4:** Comparison of the AI prediction results according to initial treatment applied, reason for consultation, and sex.

Subgroup	Sensitivity	Specificity	PPV	NPV	Accuracy
According to initial treatment applied					
Anti-inflammatory treatment before surgery (*n* = 51)	82.22%	40.00%	80.43%	42.86%	71.67%
Follow-ups without anti-inflammatory treatment before surgery (*n* = 100)	94.38%	52.63%	75.68%	85.71%	78.08%
Immediate surgical treatment (*n* = 49)	87.18%	33.33%	77.27%	50%	72.22%
According to reason for consultation					
Physical examination (*n* = 153)	92.42%	45.83%	75.78%	76.74%	75.98%
Appearance of symptoms (*n* = 47)	80.49%	60.00%	84.62%	52.94%	75.00%
According to sex					
Male (*n* = 81)	89.86%	46.15%	74.70%	72.00%	74.07%
Female (*n* = 119)	89.42%	50.00%	79.49%	68.57%	76.97%

With regard to sex, the AI system correctly predicted 80 (62 malignant and 18 benign) out of the 108 male cases, and 117 (93 malignant and 24 benign) out of the 152 female cases, showing no significant difference (*p* > 0.05) in predictive accuracy (Table 4).

### AI prediction results according to adenocarcinoma subtype

There were 87 benign cases and 173 malignant cases (including AAH) in the dataset. Stratification by pathologic subtype showed that the AI system correctly predicted all six cases of AAH, 30 out of the 33 cases of AIS, 19 out of the 20 cases of MIA, 98 out of the 107 cases of IA, none of the four cases of squamous cell carcinoma, and all of the three cases of mucinous adenocarcinoma. There were no significant differences (*p* > 0.05) in the predictive accuracy between the subgroups according to adenocarcinoma subtype or TNM staging (Table 5).

**Table 5:** Comparison of the AI prediction results according to adenocarcinoma subtype.

Subgroup	Accuracy
Pathologic type	
AIS (*n* = 33)	90.90%
MIA (*n* = 20)	95.00%
IA (*n* = 107)	91.59%
TNM staging	
Tis (*n* = 36)	94.44%
T1 (*n* = 123)	88.62%
T2 (*n* = 6)	83.33%
T3 (*n* = 6)	100%
T4 (*n* = 2)	50%

## Discussion

Lung cancer ranks first in both incidence and mortality rates among all malignant tumors in China due to the aging of the population, as well as the environment, smoking, and genetic factors (20). Pulmonary nodules are a major manifestation of early-stage lung cancer, and LDCT is recommended as the principal test for pulmonary nodule detection and lung cancer screening, since it can reduce the lung cancer mortality rate by 20% in high-risk individuals without symptoms (21). However, radiologists are faced with the dilemma of misdiagnoses caused by large volumes of data from initial screenings and re-examinations (22). An AI imaging diagnostic software with stable performance, high repeatability, and fast speed in making comparisons, can help doctors to considerably enhance the sensitivity of diagnosis, reduce the labor burden, and lower the human error rate (23). In addition to the prediction of benignity and malignancy of the pulmonary nodules, the AI system demonstrates prediction efficiency of prioritization in the subgroups with different clinical characteristics, and can even assist clinicians in prioritizing between the types of pulmonary lobectomy to be used by providing a comprehensive, objective analysis integrating the distribution of the nodules, tumor grade, size, and shape (24).

Different types of software vary in sensitivity and specificity due to different algorithms used. According to the research by Li et al. (22), the deep learning-based computer-aided diagnosis (DL-CAD) system detected 700 nodules with a sensitivity of 86.2% (95% CI, 84.1–88.8%; *p* < 0.001), and 96.5% (95% CI, 93.4–99.5%) for nodules ≥5 mm in diameter. Wan et al. (25) applied a vessel-suppression function and a deep-learning-based computer-aided-detection (*VS*-CAD) analyzer to distinguish malignant from benign nodules, and achieved a sensitivity of 93.6%, with a specificity of 39.3%. The study by Setio et al. (26) showed that a pulmonary nodule diagnostic system using multi-view convolutional neural networks (ConvNets) reached a high true-positive rate of 85% in the judgment of malignancy. Yoo et al. (27) assessed the performance of a deep learning-based nodule detection algorithm, achieving a sensitivity and specificity of 86.2% (95% CI, 77.8–94.6%) and 85.0% (95% CI, 81.9–88.1%), respectively. In this study, we conducted a retrospective validation of σ-Discover/Lung, a well-trained model with high sensitivity and specificity, by examining its prediction of benign and malignant pulmonary nodules for cases with surgical treatment performed during the period from January 2018 to April 2021 in the Affiliated Zhongshan Hospital of Dalian University. Each AI outcome was expressed as a percentage as the prediction of malignancy risk. Results showed that the AI system reached a 100% lesion detection rate for the pulmonary nodules with surgical treatment, missing none of the 260 cases. Its accuracy in predicting the benignity and malignancy of nodules measuring 5–30 mm in diameter was 75.77%, with a sensitivity of 89.60%, and specificity of 48.28%. PPV and NPV were 77.50 and 70.00%, respectively, and the AUC was 0.755, which confirmed that the AI model could be applied for the judgment of benign and malignant pulmonary nodules, and is more valuable in the prediction of malignant nodules.

In general, the smaller the pulmonary nodules measure on the chest CT, the more difficult they are for accurate prediction. In the study of Mehta et al. (28), a diameter of 5 mm was regarded as the positive cutoff value for pulmonary nodules, and their results showed that the malignancy rate was 15.3% for nodules measuring more than 10 mm in diameter, while for nodules measuring 5–10 mm it was 1.3%, and for nodules measuring less than 5 mm, only 0.4% (29). Nevertheless, it is necessary to deal with pulmonary nodules smaller than 10 mm with caution (30), as they are exactly within the most difficult size range for clinical judgment, and early diagnosis of malignant nodules measuring 5–10 mm is essential for timely surgery, smaller resection area, and better prognosis (31). In this study, the cases were classified into three subgroups based on nodule diameter (5–10, 10–20, and 20–30 mm) and the AUC was 0.778, 0.771, and 0.686, respectively, which demonstrated that the AI system had fairly good diagnostic effectiveness for all the pulmonary nodules measuring 5–30 mm in diameter, and especially for the ones measuring 5–10 mm (both inclusive) in diameter.

Based on imaging density, pulmonary nodules can be divided into solid nodules and subsolid nodules, with the latter being further divided into pure ground-glass opacity nodules (pGGNs) and mixed ground-glass opacity nodules (mGGNs) (32). According to research articles, subsolid nodules are more commonly detected in Chinese individuals compared with westerners, with a higher proportion being ground-glass opacity (GGO) (33, 34). The distribution of nodules included in the datasets reported in China and abroad also show significant differences (35, 36), with the latter mainly presenting as solid nodules and fewer as subsolid ones (37–39), and yet the malignancy rate is higher for subsolid nodules (40). This indicates that AI models trained on a foreign dataset are not necessarily fit for the diagnosis of pulmonary nodules in China. This study applied an AI model trained on a domestic dataset and examined its performance in diagnosing 260 cases confirmed by surgical pathology. The system did indeed show an advantage in judging subsolid nodules, as its predictive accuracy was higher for subsolid nodules than solid ones (80.54 vs. 66.67%, *p* < 0.05), with a sensitivity of 93.14% and PPV of 83.19%. But there no significant differences between the pGGNs and mGGNs groups, the result might be caused by the data bias of subsolid nodules in this study. Classification by nodule position showed that the AUC for the model was 0.677, 0.758, 0.744, 0.982, and 0.725, respectively, for the nodules in the left upper lobe, left lower lobe, right upper lobe, right middle lobe, and right lower lobe. The AI diagnostic effectiveness was highest for the nodules in the right middle lobe, followed by those in the left lower lobe, which differed from the study results of Horeweg et al. (41), in which the malignancy detection rate was highest in the right upper lobe. The discrepancy might be caused by the limited number of benign cases in this study.

Many cases of early-stage lung cancer have been detected during physical examinations before symptoms appear (42). In this study, malignant nodules accounted for 76.30% of the cases (without symptoms) identified during physical examinations, suggesting that it would be feasible to quickly improve the clinical diagnostic effectiveness for this subgroup by applying AI. On the other hand, there were no significant differences in the AI predictive accuracy between the cases identified during physical examinations and the cases identified during consultations for respiratory tract symptoms, or between the subgroups according to TNM staging, which could be explained by data bias, as the majority of the cases included in this study were at Stage I.

Lung cancer incidence in women has seen a continuing rise (43), among which adenocarcinoma accounts for the majority of cases and mainly presents as peripheral nodules on CT imaging (44). According to the current study, the malignancy rates for males and females were similar (74.07 vs. 76.97%, *p* > 0.05). Nor did the AI predictive accuracy show any significant difference between male and female (*p* > 0.05).

Different pathologic types of pulmonary nodules vary in imaging characteristics. Numerous studies have confirmed that most long-term existed GGNs in the lung are mostly early lung adenocarcinoma or their precancerous lesions (45). In this study, the dataset mainly consisted of adenocarcinoma cases, while squamous cell carcinoma was rare. The former included 33 cases of AIS, 20 cases of MIA, and 107 cases of IA, and the AI prediction accuracy was 90.90, 95, and 91.59%, respectively, without a significant difference in accuracy (*p* > 0.05). Similarly, Zhao et al. (46) found no significant differences in the AI predictive accuracy of tumor invasiveness between AAH-AIS, MIA, and IA, probably because the subtle differences in imaging characteristics among the pathologic subtypes of adenocarcinoma were difficult to acquire by the deep neural networks, and an imbalanced or inadequate training dataset could also restrict the diagnostic effectiveness of the system. In contrast, in the study of Shao et al. (47), the effectiveness of applying the maximum standardized uptake value (SUV_max_) to distinguish between pathologic subtypes of pulmonary adenocarcinoma showed statistically significant differences, and Le et al. (35) concluded that the quantitative measurement using weighted random forest classifier had fairly good performance in the classification of pulmonary adenocarcinoma subtypes, both of which suggest that it would be feasible to enhance the AI predictive accuracy for pulmonary nodules by further intelligent optimization of the model.

In summary, the AI system demonstrated fairly good accuracy, sensitivity, and positive predictive value in the prediction of benignity and malignancy of the pulmonary nodules in this study, which could contribute to improving efficiency in clinical practice and to reducing missed diagnoses. It had better diagnostic effectiveness in predicting the malignancy risk for the small nodules measuring 5–10 mm in diameter, which is difficult for humans to determine. With regard to the different clinical characteristics, the AI model showed significant differences in the predictive accuracy between the subgroups according to the nodule position, and nodule density, suggesting it has an advantage in the prediction for these clinical subgroups. Generally speaking, the sensitivity of the AI prediction was high but the specificity was comparatively low in this study, which is a common issue that has needed to be addressed since the application of AI in this medical field (22). In addition, we collected only a limited number of pulmonary nodule cases with pathologic diagnosis as per the “gold standard,” and certain biased data, such as far fewer benign nodules than malignant nodules, affected the specificity of the model, resulting in high positive predictive value and low negative predictive value. It has been determined by many factors that at present the effectiveness of applying AI for the detection of pulmonary nodules, and the differentiation between benignity and malignancy, has not met clinical expectations, and larger datasets need to be used for the training of deep neural networks. As for future research, we believe that improvement in the AI diagnostic effectiveness can be made possible by expanding the labeled database, increasing the amount of validation samples (especially with a larger number of benign cases), and training the model on a dataset with more comprehensive clinical information about the patients in addition to their lung conditions, alongside developments in the field of deep learning. An independent validation study using datasets collected from other institutes, regions, and races would be of high clinical importance. It is also worthy of further study whether the AI model has a significant predictive advantage for subgroups classified by other clinical characteristics, how to realize more accurate risk stratification for GGNs, and how to assist doctors in the clinical management of pulmonary nodules, the choice of types of surgery, and the assessment of prognosis.

## Data availability statement

The original contributions presented in the study are included in the article/supplementary material; further inquiries can be directed to the corresponding author.

## Ethics statement

The studies involving humans were approved by Ethics Committee of Affiliated Zhongshan Hospital of Dalian University (approval no. 2019068). The studies were conducted in accordance with the local legislation and institutional requirements. The participants provided their written informed consent to participate in this study.

## Author contributions

LZ: Conceptualization, Writing – original draft, Writing – review & editing, Data curation, Project administration, Validation, Visualization. YS: Data curation, Formal analysis, Investigation, Methodology, Project administration, Validation, Writing – review & editing. GC: Data curation, Investigation, Methodology, Writing – review & editing. ST: Data curation, Writing – review & editing. QZ: Investigation, Writing – review & editing. JW: Data curation, Writing – review & editing. CB: Visualization, Writing – review & editing, Supervision. DY: Conceptualization, Data curation, Funding acquisition, Investigation, Methodology, Validation, Visualization, Writing – original draft, Writing – review & editing.
